# Anticoagulation Management With Coumarinic Drugs in Chilean Patients

**DOI:** 10.1177/1076029619834342

**Published:** 2019-03-17

**Authors:** Elena Nieto, Marcelo Suarez, Ángela Roco, Juan Carlos Rubilar, Francisca Tamayo, Mario Rojo, Gabriel Verón, Juliana Sepúlveda, Fanny Mejías, Patricio Salas, María Góngora, Patricio Andrade, Alicia Canales, Jorge Carabantes, Daniela Cruz, Emma Contreras, Daniela Pavez, Paulina Charo, Gabriela Bravo, Juan Calderón, Carlos Gallardo, Patricia Vega, Luis A. Quiñones

**Affiliations:** 1 San Juan de Dios Hospital, Santiago, Chile; 2 Laboratory of Chemical Carcinogenesis and Pharmacogenetics, Department de Basic-Clinical Oncology, Faculty of Medicine, University of Chile, Santiago, Chile; 3 Escuela de Bioquímica Facultad de Ciencias de la Vida, Universidad Andrés Bello, Santiago, Chile; 4 Western Metropolitan Health Service, Santiago, Chile; 5 Félix Bulnes Cerda Hospital, Santiago, Chile; 6 San José de Melipilla Hospital, Santiago, Chile; 7 Health Reference Center “Dr. Salvador Allende G”, Santiago, Chile; 8 Talagante Hospital, Santiago, Chile; 9 Peñaflor Hospital, Santiago, Chile; 10 Curacavi Hospital, Santiago, Chile

**Keywords:** acenocoumarol, warfarin, thrombosis, safety, telemedicine

## Abstract

Warfarin and acenocoumarol are used in various cardiovascular disorders to improve the prognosis of patients with thromboembolic disease. However, there is a lack of substantial efficacy and safety data on antithrombotic prophylaxis in several countries, particularly in Latin America. The aim of this study was to provide information about the efficacy of anticoagulants in Chilean patients. Data were collected from databases of the Western Metropolitan Health Service, Santiago, Chile. We identified 6280 records of patients receiving anticoagulant treatment. The three most common diagnoses were rhythm disorder (43.7%), venous thrombosis (22%), and valvular prosthesis (10.7%). The majority of patients (98.5%) received acenocoumarol while 1.5% of patients received warfarin, at weekly therapeutic doses of 13.6 mg and 30.4 mg, respectively. For total diagnoses, the median time in the therapeutic range was 50%. However, better results, 66.7%, were observed when a telemedicine strategy was used only in Santiago Province. Our findings emphasize that in Chile, where the number of patients receiving anticoagulant treatment increases every year, telemedicine, by committed teams, improves the use of oral anticoagulants and is able to increase quality indicators of anticoagulant treatment care.

## Introduction

Cardiovascular disease (CVD) is the leading cause of death worldwide. In 2015, an estimated 17.7 million people died from CVD (31% of all global deaths). Of these deaths, an estimated 7.4 million were due to coronary heart disease and 6.7 million were due to stroke. The estimated number of deaths is projected to increase to 23.6 million per year by 2030.^1,2^ Most CVDs can be prevented by addressing behavioral risk factors such as tobacco use, unhealthy diet and obesity, physical inactivity, and abuse of alcohol, using population-wide strategies.^1,2^ In Chile in 2012, the overall rate of death attributable to CVD was 156.2 per 100 000 Chileans: 160.2 for men and 152.2 for women. These figures have continued to rise in recent years,^3^ and presently, the Chilean public health goal for the decade 2011 to 2020 is to increase both CVD survival and the proportion of people with controlled arterial hypertension.^4^ Many patients who survive a cardiovascular (CV) event are prescribed an oral anticoagulant to prevent future thromboembolic events. The latest data published by the Chilean Department of Statistics and Health Information, for 2017, establishes that the number of visits to the emergency services exceeds 6000 per week for patients with CVD.^5^

Coumarin derivatives (vitamin K antagonists [VKA]), such as warfarin and acenocoumarol, are used in various disorders including deep venous thrombosis, pulmonary embolism, atrial fibrillation (AF), and artificial heart valves. They have improved the prognosis of patients with thromboembolic disease. An individual’s response to coumarins depends on several factors. The nongenetic factors include age, gender, body mass index, diet, and interacting drugs.^6^ Among the genetic factors, the cytochrome P450 system and vitamin K epoxide reductase complex subunit 1 play a key role in drug metabolism.^7,8^ Complications from inappropriate warfarin dosing are among the adverse events most frequently reported to the US Food and Drug Administration and one of the most common reasons for emergency room visits.^8^ Bleeding is the most common complication of antithrombotic therapy and predicts subsequent CV events. Although the mechanisms linking bleeding with an increased risk of CV events after bleeding remain poorly understood, prevention of bleeding can be expected to avoid related morbidity and mortality.^9^

The weekly therapeutic dose (WTD) of coumarins is calculated using the prothrombin time, expressed as international normalized ratio (INR). The range of anticoagulation depends on the etiology: in each control, the INR is measured and the dose of coumarin is adjusted accordingly. It can take around 6 months or more to achieve an adequate dose in some patients. The 2 most commonly reported parameters for INR control are frequency in range (FIR), also known as proportion of INRs in the therapeutic range and number of tests in range, and the proportion of time in the therapeutic range (TTR).^10,11^ In a study on the prognostic role of TTR, the most widely used indicator of the quality of anticoagulation therapy, it was observed that of patients treated with coumarins, the rates of cerebrovascular accident/systemic embolisms and bleeding were significantly lower in patients who achieved a TTR greater than 65%, and that this also translated to lower mortality.^12^ The National Institute for Health and Care Excellence (NICE) recommends that the TTR must be greater than 65%,^13^ the European Society of Cardiology recommends a target TTR of at least 70%,^14^ and the Asia Pacific Heart Rhythm Society recommends a TTR of at least 60%^15^ for optimal VKA control.

In Chile, oral anticoagulant treatment is performed by medical specialists in high and medium complexity hospitals, unlike in other countries of the region where it is performed by general practitioners. There are very few studies of patients in Chile who are undergoing treatment with coumarin derivatives. One of these studies, from Antofagasta, with 135 patients (with an average age of 62.1 years) undergoing valve replacement. Of the patients, 77.4% were women, 90% had a mechanical prosthesis, and 60% had an INR in the indicated range.^16^ A recently published study using the Global Anticoagulant Registry in the Field (GARFIELD-AF) registry in Chile included 971 patients with AF, recruited both in public hospitals (85%) and private clinics (15%). The 70% of patients who were treated with VKA had a median TTR of 40%. As an adverse event, 36 patients presented with cerebrovascular accidents.^17^

The Western Metropolitan Health Service (WMHS) includes urban and rural population and is divided into 3 provinces: Santiago, Melipilla, and Talagante. Transfer/mobilization time from rural and urban areas varies between 2 to 3 hours, which increases the lack of patient attendance to their control in hospitals. Thus, to improve the adherence to anticoagulant therapy, the INR capillary sample collection was implemented at the primary care center closer to their home and specialist consultation through telemedicine using videoconference licenses delivered by the Ministry of Health. Therefore, the aim of this study was to provide more complete information about the characteristics of Chilean patients with coumarinic anticoagulant treatment, management strategies, and clinical results and to evaluate the impact of the telemedicine strategy on the quality of this anticoagulant treatment.

## Methods

### Study Design

A retrospective study of patients using controlled warfarin (Coumadin, Merck, Darmstadt, Germany) or acenocoumarol (Coarol, Andrómaco, Santiago, Chile) antithrombotic therapies in the WMHS in the Santiago, Melipilla, and Talagante provinces of Chile. The INR measurement was performed with a capillary sample using CoaguChek equipment (Roche Diagnostic, Mannheim, Germany).

### Ethics Statement

The research was authorized by the ethics committee of the WMHS (N° 036-2017).

### Data Collection

Patient data were obtained from clinical centers and managed with the statistical module of the anticoagulant treatment dosing software (TAONet, Roche Diagnostics, Mannheim, Germany) from February 06, 2010, to August 18, 2017.

### Oral VKA Anticoagulant Treatment

The initial dose was one 4-mg tablet of acenocoumarol or one 5-mg tablet of warfarin on day 1. On day 2, the dose was decreased to 50% (half a tablet). The INR was controlled on day 3; thus, if the INR was higher than 1.8, the dose was again reduced by 50%, and patients were checked in 2 days for medical control to adjust the dose according to the INR results.

### Calculation of FIR and TTR

The target range of INR for this study was 2.0 to 3.0, based on the recommendations from international guidelines.^14^ Patients with other INR ranges were excluded for these indicators.

Population-level FIR was calculated as the percentage of the total INR readings, in the range, for all patients. Patient-level TTR was estimated by assigning INR values to each day between consecutive INR readings by linear interpolation, as described by Rosendaal et al.^10^ The calculation of these indicators was performed by trimesters, based on the recommendations from international guidelines.^13^ Trimester I includes January to March, trimester II includes April to June, trimester III includes July to September, and trimester IV includes October to December.

#### Telemedicine

A mobile assistance device was implemented in each commune and a license for videoconferencing using the MINSAL network was installed in the specialist medical care box at the Hospital Anticoagulant Polyclinic. The determination of INR was performed on a Roche Coagucheck device connected to an Anticoagulant Treatment software (TAONet; Roche Diagnostics). The specialist doctor made the consultation via Telemedicine and the dosage (prescription) of the anticoagulant drug was delivered in the commune where the patient was treated.

### Statistical Analysis

To evaluate the normality of the data, a nonparametric test was used, specifically the Kolmogorov–Smirnov test (K-S) and then student *t* test.

## Results

In the Western area of the Metropolitan Region of Santiago de Chile, 6280 patients undergoing anticoagulant treatment attended the Health Service: 61.9% in the province of Santiago, 14.8% in the province of Melipilla, and 23.3% in the province of Talagante (Table 1).

**Table 1.** Patient Characteristics.

Province	Santiago	Melipilla	Talagante	WMHS
Total	3886	927	1467	6280
Age (years) ± SD	67.3 ± 14.5	68.4 ± 14.4	69.0 ± 14.4	68.3 ± 14.5
Men (%)	1803 (53.2)	507 (54.7)	748 (51)	3056 (48.7)
Age (years) ± SD	67.7 ± 13.9	69.2 ± 13.2	68.3 ± 13.9	68.2 ± 13.7
Women (%)	2086 (46.8)	420 (45.3)	719 (49)	3224 (51.3)
Age (years) ± SD	66.8 ± 15.1	67.6 ± 15.8	69.8 ± 14.9	68.5 ± 15.1
Treatment with acenocoumarol				
Total (%)	3858 (99.3)	918 (99.0)	1411 (96.2)	6187 (98.5)
Weekly therapeutic dose				
Men ± SD	13.6 ± 7.0	14.8 ± 7.9	14.3 ± 7.6	13.9 ± 7.3
Women ± SD	13.0 ± 7.3	13.8 ± 7.7	13.5 ± 7.7	13.2 ± 7.4
Total Average dose ± SD	13.3 ± 7.2	14.3 ± 7.8	13.9 ± 7.7	13.6 ± 7.4
Therapeutic INR range				
2.0-3.0 (%)	3255 (84.4)	819 (89.2)	1356 (96.1)	5431 (87.8)
3.0-4.0 (%)	56 (1.5)	2 (0.2)	4 (0.3)	62 (1.0)
Other (%)	547 (14.2)	97 (10.6)	51 (3.6)	694 (11.2)
Duration of treatment				
<12 months (%)	591 (15.4)	127 (13.8)	225 (15.9)	943 (15.2)
12-24 months (%)	700 (18.1)	183 (19.9)	282 (20.0)	1165 (18.8)
25-36 months (%)	469 (12.1)	106 (11.5)	220 (15.6)	795 (12.8)
>36 months (%)	2098 (54.3)	502 (54.7)	684 (45.5)	3284 (53.1)
Treatment with Warfarin				
Total (%)	28 (0.7)	9 (1.0)	56 (3.8)	93 (1.5)
Weekly therapeutic dose				
Men ± SD	20.0 ± 10.3	41.7 ± 11.7	31.7 ± 15.7	30.2 ± 15.4
Women ± SD	27.1 ± 20.4	36.5 ± 11.4	31.5 ± 12.9	30.4 ± 15.9
Total Average dose ± SD	25.4 ± 18.5	38.2 ± 11.1	31.6 ± 14.1	30.4 ± 15.6
Therapeutic range				
2.0-3.0 (%)	17 (60.7)	7 (77.8)	8 (14.3)	32 (34.4)
3.0-4.0 (%)	1 (3.6)	0 (0)	13 (23.2)	14 (15.1)
Other (%)	10 (35.72)	2 (22.2)	35 (62.5)	47 (50.5)
Duration of treatment				
<12 months (%)	5 (17.9)	0 (0)	1 (1.8)	6 (6.5)
12-24 months (%)	7 (25)	0 (0)	6 (10.7)	13 (14.0)
25-36 months (%)	2 (7.1)	1 (11.1)	3 (5.4)	6 (6.5)
>36 months (%)	14 (50)	8 (88.9)	46 (82.1)	68 (73.1)

Abbreviations: INR, international normalized ratio; SD, standard deviation; WMHS, Western Metropolitan Health Service.

In Santiago, of a total of 3886 controlled patients with an average age of 67.3 ± 14.5 years, 51.3% were women and 48.7% were men, and 99.3% were treated with acenocoumarol at an average WTD of 13.3 ± 7.2 mg. In the province of Melipilla, 927 patients with an average age of 68.4 ± 14.4 years (54.7% men and 45.3% women) were controlled; 99% used acenocoumarol at an average WTD of 14.3 ± 7.8 mg. Finally, in the Talagante province, 1467 patients with an average age of 69.8 ± 14.9 years were controlled (51% men and 49% women); 96.2% used acenocoumarol with a WTD of 13.9 ± 7.7 mg (Table 1).

A range of anticoagulation with INR between 2.0 and 3.0 was assigned to 87% of the patients. Of the patients, 66% were treated with acenocoumarol for more than 25 months, 18.8% for 12 to 24 months, and only 15.2% were treated for less than 12 months. The WTD of acenocoumarol was 13.6 ± 7.4 mg: This dose was highest in Melipilla, at an average of 14.3 ± 7.8 mg, and especially in men (14.8 ± 7.9 mg); in Talagante, patients were administered the second highest average dose, at 13.9 ± 7.7 mg, again it was higher in men (14.3 ± 7.6 mg). Only 1.5% of patients used warfarin, mainly due to its high cost and the lack of bioequivalent drugs in Chile. The lowest use of warfarin was in Santiago (0.7%). Of the patients on warfarin, 73.1% were treated for more than 36 months with an average WTD of 30.4 ± 15.6 mg; the highest average dose was in Melipilla, at 38.2 ± 11.1 mg, again this was higher in men at 41.7 ± 11.7 mg (Table 1).

The most frequent diagnosis for the use of oral anticoagulant treatment was rhythm disorders, at 43.7%. These disorders include complete arrhythmia, AF, atrial flutter, definitive pacemaker, and node disease. The highest percentage of rhythm disorders was observed in Talagante, at 52.7%, followed by Melipilla at 50.2%. The second most frequent diagnosis was venous thrombosis, at 22%, and this includes deep vein thrombosis/pulmonary embolism, superficial venous thrombosis, phlebitis and thrombophlebitis of lower extremities, and lower extremity varicothrombosis. Again, this diagnosis was higher in patients from Talagante (24.3%). The third most frequent diagnosis corresponded to mechanical or biological valvular replacement (10.7%) and was the highest in the province of Santiago (13.2%; Table 2). The most frequent comorbidities were hypertension (18.6% of patients) and metabolic disorders, including diabetes and obesity (15.6% of patients; Table 2).

**Table 2.** Primary Diagnoses and Greater Comorbidities of Patients.

Primary Diagnoses	Santiago, n (%)	Melipilla, n (%)	Talagante, n (%)	WMHS, n (%)
Rhythm disorder	1507 (38.8)	465 (50.2)	773 (52.7)	2745 (43.7)
Venous thrombosis	809 (20.8)	218 (23.5)	357 (24.3)	1384 (22.0)
Biological valvular prosthesis	515 (13.2)	74 (8.0)	84 (5.7)	673 (10.7)
Stroke	142 (3.7)	81 (8.8)	116 (7.9)	339 (5.4)
Cardiomyopathies	43 (1.1)	38 (4.1)	47 (3.2)	128 (2.0)
Hereditary-acquired thrombophilia	88 (2.3)	31 (3.3)	17 (1.2)	136 (2.2)
Occlusive arterial disease	59 (1.5)	15 (1.6)	48 (3.3)	122 (1.9)
Congenital valve disease	51 (1.3)	3 (0.3)	15 (1.0)	69 (1.1)
Venous thromboembolism (VTE) prevention	12 (0.3)	2 (0.2)	1 (0.07)	15 (0.2)
Other pathologies	660 (17.0)	0 (0)	9 (0.6)	669 (10.7)
Comorbidities				
Hypertension	625 (16.1)	238 (25.7)	301 (20.4)	1164 (18.6)
Metabolic disorders	647 (16.6)	135 (14.6)	194 (13.2)	976 (15.6)
Cardiomyopathies	471 (12.1)	168 (18.2)	158 (10.2)	797 (12.7)

Abbreviation: WMHS, Western Metropolitan Health Service.

To calculate the therapeutic range (FIR) and the TTR indicators, the first 3 controls of the patient were excluded, in addition to the INR values in posthospitalization or posttreatment control or other clinical situation that is known to interfere with the INR value. Both the percentage of INR in the therapeutic range (FIR >50%) and the TTR (>65%) are below the percentages considered a good control of anticoagulant treatments. The median of the TTR in the last trimester of 2017 was 50 for the WMHS: for the provinces of Santiago, Melipilla, and Talagante, the median was 50, 40, and 33, respectively. When considering only AF or diagnosis of thrombosis, the median values of TTR remain the same as for all diagnoses (Table 3).

**Table 3.** Indicators Median of Time in Therapeutic Range (TTR) Range 2.0 to 3.0 and Population-Level FIR: Percentage of the Total INR Readings That Were in Range for Total of Patients.

Province	2017 Trimesters				Total Province
	I	II	III	IV	
FIR					
Santiago	49.5	48.2	49.5	50.1	49.4
Melipilla	46.6	47.2	47.2	48.4	47.3
Talagante	44.1	42.9	42.7	45.3	43.7
WMHS	47.8	46.8	47.5	48.7	47.7
TTR					
Santiago	40	40	50	50	50
Melipilla	33	40	40	40	40
Talagante	33	33	33	33	33
WMHS	40	40	50	50	50

Abbreviations: FIR, frequency in range; INR, international normalized ratio; TTR, time in the therapeutic range; WMHS, Western Metropolitan Health Service.

In 2014, in an aim to improve the quality of anticoagulant treatment, a pilot telemedicine care study was started between the San Juan de Dios Hospital, located in the province of Santiago, and the Hospital de Curacaví, located 66 km away. An improvement in both the FIR and TTR indicators was observed.^18^ Since 2014, telemedicine care for anticoagulant treatment has increased, and as shown in Table 4, both the FIR and the TTR have better results when using telemedicine. The greatest impact is observed in the province of Santiago both in FIR (*P* value .01) and in TTR (*P* value .016).

**Table 4.** Results of the FIR and TTR Indicator in Patients Face-to-Face Care Versus Telemedicine Care of 2017.

		FIR			TTR		
Province	Quarters	Face to Face	Telemedicine	*P* Value^a^	Face to Face	Telemedicine	*P* Value^a^
Santiago	I	49.5	56.0	**.001**	40	63.3	**.016**
	II	48.2	53.3		40	50	
	III	49.5	56.8		50	63.3	
	IV	50.1	55.9		50	66.7	
Melipilla	I	46.6	44.2	.299	33	40	.554
	II	47.2	51.5		40	33	
	III	47.2	48.7		40	40	
	IV	45.9	51.1		40	50	
Talagante	I	44.1	44.9	.275	33	33	.356
	II	42.9	43.9		33	33	
	III	42.7	44.0		33	33	
	IV	45.3	49.2		33	50	

Abbreviations: FIR, percentage of the total INR readings that were in range for total of patient; INR, international normalized ratio; TTR, time in therapeutic range.

Significant values (*p*<0.05) are in bold.

^a^ *P* value: student *t* test.

Finally, when comparing dosage and ethnicity, the average daily dose of warfarin is similar in both Caucasians and the Spanish population, whereas the dose of acenocoumarol is much lower in Caucasians than in the Spanish population^7,19^ (Table 5).

**Table 5.** Average Daily Dose in Patients With Oral Anticoagulant Treatment According to Ethnicity and Type of Coumarin.^7,20^

	Warfarin					Acenocoumarol	
	African American	Caucasian	Asian	Chile (This Study)	Spanish	Spanish	Chile (This Study)
Average dose (mg/d)	5.2 ± 1.7	4.3 ± 2.2	2.7 ± 1.1	4.3 ± 2.2	4.0 ± 1.1	2.0-3.0	1.9 ± 1.1

## Discussion

In the Western area of the Metropolitan Region of Santiago de Chile, 98.5% of patients on anticoagulant treatment use acenocoumarol at an average WTD of 13.6 ± 7.4 mg. Appropriate dosing of coumarins is difficult to establish due to significant interindividual variability in the dose required to obtain stable anticoagulation.^21^

Although warfarin and acenocoumarol are similar, the recommended doses are different and there are differences in their pharmacokinetics and pharmacodynamics, as well as in the influence of genetics and other factors. In Chilean patients, the difference in the dose of acenocoumarol compared to that in Spanish patients could be explained by a different frequency in the polymorphisms of metabolizing enzymes of VKA (CYP2C9*2, CYP2C9*3) or of enzymes that participate in the vitamin K cycle, such as vitamin K epoxide reductase complex subunit 1 (VKORC1).^21,22^ In a Spanish study, it was observed that the pharmacogenetic algorithm correctly predicted the real stable dose in 59.8% of the cases, whereas only 37.6% were correctly predicted by the clinical algorithm.^21^

It is important to highlight that the consumption of green vegetables (rich in vitamin K) is very variable worldwide, between 100 g/d in undeveloped countries up to around 450 g/d in developed countries.^23^ This was a factor that was controlled in this study. However, it has been reported that the consumption of green vegetables and fruits by Chilean people is around 235 g/d for women and 220 g/d for men. This diet not only includes tomato, lettuce, and carrots but also a smaller proportion of green vegetables with high vitamin K content. Therefore, this could be a factor in the low quality of anticoagulation of our patients.^23^

The medical management of patients with AF with oral anticoagulants differs across Europe: for example, in one study, the proportion of patients taking VKA varied between 86.0% (in France) and 71.4% (in Italy). Warfarin was used predominantly in the United Kingdom and Italy (74.9% and 62.0%, respectively), phenprocoumon in Germany (74.1%), acenocoumarol in Spain (67.3%), and fluindione in France (61.8%). The major sites for INR measurements were biology laboratories in France, anticoagulation clinics in Italy, Spain, and the United Kingdom, and physicians’ offices or self-measurement in Germany. Time in the therapeutic range ranged from 70.3% in Spain to 81.4% in Germany. While the type and half-lives of VKA as well as the mode of INR surveillance differed, overall quality of anticoagulation management by TTR was relatively homogenous in patients with AF across countries.^19,24^

This study shows that for all diagnoses, the median TTR is 50(%), lower than that recommended.^13^ Our results for AF are similar to those found by Corbalan et al, which also includes patients who are recruited in the private health system.^17^ However, when the telemedicine strategy is used, the TTR indicator increases, achieving the value recommended by NICE.^13^ This can probably be explained by the local telemedicine team (formed by nurses, nutritionists, and pharmaceutical chemists) reinforcing the indications given in the control by the specialist doctor, both to the patient and to family, as well as an improvement in adherence to the therapy derived from an easier access to the control perceived by the patients. This meant lower risk of thrombosis or hemorrhage in these patients, thus avoiding visits to the emergency department and hospitalizations.

A main limitation of the present work should be noted. This study is lacking data on adverse medical events such as mortality (CV and non-CV), stroke, and major bleeding. This is truly important to evaluate efficacy and safety of this strategy.

## Conclusion

This is the first study of oral coumarinic anticoagulant treatment across a population in Latin America, with the results showing that there are differences between the effective doses of anticoagulants in Chilean patients of different ethnic groups. Our patients present a median TTR of 50, less than that considered optimal for reducing the rates of stroke/systemic embolisms, bleeding, and mortality. Telemedicine, by committed teams, increases the quality indicators of anticoagulant treatment care (in the case of the province of Santiago, the median TTR increased to 66.7, *P* value .016). This validates the use of telemedicine as a clinical tool over long distances, bringing the specialist closer to communities far from complex hospital centers. It is accepted, valued, and very highly regarded by its users.^18^ Additionally, pharmacogenomics testing may provide added clinical value to the use of acenocoumarol and warfarin in the Chilean population.
